# Preclinical Medical Students' Perspectives and Experiences With Structured Web-Based English for Medical Purposes Courses: Cross-Sectional Study

**DOI:** 10.2196/65779

**Published:** 2025-03-27

**Authors:** Radhakrishnan Muthukumar, Isaraporn Thepwongsa, Poompong Sripa, Bangonsri Jindawong, Kamonwan Jenwitheesuk, Surapol Virasiri

**Affiliations:** 1 Academic Affairs, Faculty of Medicine, Khon Kaen University, Khon Kaen, Thailand; 2 Department of Community, Family and Occupational Medicine, Faculty of Medicine, Khon Kaen University, 123 Mittraparb Road, Khon Kaen, 40002, Thailand, 66 43363588; 3 Inverkeithing Medical Group, Inverkeithing, United Kingdom

**Keywords:** English for medical purposes, online course, online learning, online education, medical students, medical school, online, online learners, perspectives, English, English language, medical research, educational method, lesson, course, instructional designs, English for medical professional, EMP, barriers, web-based

## Abstract

**Background:**

English for medical purposes (EMP) is essential for medical students as it serves as a foundational language for medical communication and education. However, students often undervalue its importance within the medical curriculum. Given their demanding schedules and workload, educational methods for EMP must align with their needs. Structured web-based learning offers flexibility and convenience, yet limited research has explored its exclusive application for EMP in undergraduate medical education.

**Objective:**

This study aimed to evaluate medical students’ perspectives on structured web-based EMP courses and assess their impact on medical English proficiency using objective and subjective measures.

**Methods:**

Structured web-based EMP courses were developed based on evidence-based guidelines, addressing barriers to web-based learning during development and implementation. A cross-sectional study was conducted with 535 medical students who completed these courses. Data were collected via questionnaires, the learning management system, and the Khon Kaen University Medical English Test (KKUMET), which assessed proficiency in listening, reading, writing, and speaking. Data were analyzed using descriptive statistics.

**Results:**

Of the 535 students, 452 (84.5%) completed the survey. Participants reported confidence in reading (mean 4.11, SD 0.87), vocabulary (mean 4.04, SD 0.84), and listening skills (mean 4, SD 0.89), but lower confidence in writing skills (mean 3.46, SD 1.07). The KKUMET results showed statistically significant improvements in all 4 language skills after course completion (*P*<.001). The top-rated benefits of the courses were convenience (mean 4.77, SD 0.59), sufficient instruction (mean 4.5, SD 0.85), and clear content (mean 4.41, SD 0.80).

**Conclusions:**

Structured web-based EMP courses are relevant and well received by medical students. These courses significantly improve students’ medical English proficiency, as evidenced by both subjective feedback and objective measures. Medical educators should consider integrating structured web-based EMP programs to better support students’ language proficiency in medical contexts.

## Introduction

### Background

English is the international language of medicine [1,2]. It is the dominant language used in medical research and publications [1,3]. A growing number of medical journals worldwide have been published in English [4]. Almost 90% of the world’s publications indexed in the MEDLINE database are published in the English language [3,5]. In addition, considering their high impact factors and citation scores, almost all quality research articles have been published in English-medium journals [1].

The language of medicine has shifted from Latin and Greek to English, influencing the development of modern medical terminology [3]. The use of English in a medical community involves communication with colleagues and staff, reading medical journals, giving conference presentations, or pursuing postgraduate education in English-speaking countries [1,3]. Therefore, the English language and literacy are essential in medical curricula [2,4,6].

General English focuses on overall language competency, emphasizing foundational grammar, vocabulary, and communication skills applicable to a wide range of everyday and academic contexts. English for medical purposes (EMP), on the other hand, is a specialized subset of English tailored to the medical field. It emphasizes medical terminology, reading and comprehension of medical texts and research articles, professional communication skills that are required for clinical and academic purposes, such as writing case reports, interacting with colleagues, and participating in medical discussions [7].

Approximately half of medical schools in the world use English as a medium of instruction [8]. The current status of English use with other languages in medicine is that “English is one lingua franca in medicine but speaks many tongues” [5]. This means that although medical teaching and patient interaction are in the local language, medical professionals still use medical languages with specialized vocabulary in their professional communication [3]. For students or professionals whose English is not their first language, using medical English for their professional-related activities is much more challenging and demanding than it is for those whose English is their first language [3].

The assessment of learning needs is considered fundamental to EMP curriculum design [2,4,7,9] and should be identified early [10]. The learner-centered approach is used to obtain needs from the learner’s needs assessment. The learner-centered approach empowers learners to decide what, how, and where to learn, while teachers act as facilitators [11,12]. Learning needs assessments help define program goals and specific teaching objectives [7,13], which leads to the development of lesson plans, materials, tests, assignments, and activities. Many studies have examined the need for EMP [2,9,13-15]. Medical students need to read medical literature, listen to lectures, and write clinical reports and short essays, whereas practitioners need patient interaction and conference participation [1,7]. The development of EMP courses addresses specific language challenges that hinder medical students’ learning, particularly in contexts where English is partially used as the medium of instruction, while local languages dominate communication between instructors, students, and patients [16].

Teaching an EMP course differs from teaching general English [7,14]. Instead of learning grammar and fundamental structures, the goal of learning English at this level is to apply the language to medical studies [7]. Previous studies have described the development of EMP courses, including detailed information about curriculum development processes based on students’ learning needs [2,16-18], as well as EMP courses developed for international medical graduates [18] and undergraduate medical students [2,16,17]. However, these EMP courses were taught in medical curricula in countries where English was the official language or medium of instruction. In other countries, the EMP course design has not yet been clearly explored. As a result, the requirements of EMP for medical students must be investigated [19], and EMP instruction must be tailored to the intended audience needs. In addition, many studies have focused on preparing students with linguistic tasks for their future careers [9,15]. Less is focused on the design of EMP courses aiming to improve academic performance during undergraduate studies, preclinical years in particular, where basic science knowledge is most of the content rather than clinical application.

Educational methods for EMP should also be matched with learners’ needs and preferences. Online and web-based educational methods have been adopted in medical education for the past decade [20]. Online teaching was the primary modality of instruction offered to undergraduate medical students during the COVID-19 pandemic [21]. The web-based education methods have been tested for their effectiveness [22-24]. The advantages of web-based educational methods include improving medical students’ knowledge and skills [24]. However, prior meta-analyses have demonstrated that web-based learning is just as effective as the traditional learning methods [23,24]. There are few studies on the exclusive use of web-based learning techniques in EMP. One study explored students’ readiness for internet-based learning in EMP [25] or internet- and computer-based as part of blended learning for general English [26,27]; however, none has solely used web-based educational methods in EMP courses for medical students.

### Problem Statement

Despite the growing adoption of web-based learning methods in medical education [28], limited research has explored their exclusive use in teaching EMP, especially for preclinical medical students [24]. Web-based education, proven to be as effective as traditional methods [22,23,29,30], offers unique advantages such as flexibility and accessibility [24]. However, few studies have investigated the development and evaluation of structured web-based EMP courses tailored to students in non–English-speaking environments.

Thus, this study developed an exclusive structured web-based learning program for EMP and evaluated its effectiveness. Grounded in constructivist principles, the study emphasizes active, self-directed learning. The structured web-based EMP courses were designed to enable students to construct knowledge through interactive activities, scenario-based learning, and multimedia resources. These approaches foster engagement and empower learners to take ownership of their educational journey, ensuring the content is relevant and applicable to their academic and professional needs. The guiding research question for this study was whether a structured web-based learning program exclusively for EMP would be accepted, considered relevant, and meet the satisfaction of preclinical medical students. We hypothesized that learning EMP through structured web-based courses would be both relevant and well-received by preclinical medical students. This investigation formed the basis for evaluating the course. To comprehensively evaluate the course, the research is divided into 2 parts. The first part, the present study, focuses on assessing the relevance and acceptance of structured web-based EMP courses based on students’ perceptions of the web-based learning mode. The second part examines the effectiveness of the structured web-based EMP courses in improving students’ medical English proficiency. To evaluate the first part, a cross-sectional descriptive study was conducted to assess the relevance and acceptance of the structured web-based EMP courses among participating medical students. Additionally, feedback from course instructors, gathered during the course design and development phases, was incorporated to capture their perceptions of the web-based EMP program. The findings from the second part are discussed in a separate article; however, this study included significant findings on the program’s effects on students’ medical English proficiency. The findings are intended for medical educators, curriculum designers, and policy makers in medical education, particularly those serving non–English-speaking regions.

### Our Medical School Curriculum

Our medical school recruits students directly from high school through the Thailand University Central Admission System (TCAS). TCAS, implemented in 2018, is an admission framework consisting of 5 rounds held annually. Medical schools in Thailand often adopt unique criteria and processes within TCAS, reflecting their specific admission requirements and competitive nature [31]. With 288 students enrolled each year, our medical school is one of the regional institutions in northeastern Thailand. The undergraduate medical curriculum spans 6 years, including the first 3 years focused on medical sciences and the last 3 years on clinical practice [32]. Our 2019 updated medical curriculum states that medical students must earn 259 credits over 6 years of medical school. In the first year, premedical education courses (the majority are general education and general principles for medical sciences) account for 38.5 credits. In the second and third years, preclinical education courses comprise 76.5 credits. Clinical rotations, accounting for 48 credits, are conducted in the sixth year, while clinical education courses make up 96 credits across the fourth and fifth years [33]. The majority of the lecture slides, suggested texts, teaching and learning materials, and exam questions are in English, even though the instruction at our school is in Thai.

It is mandatory for Thais to learn English from primary school till higher education [34,35]. In Thailand, the English component taught in high school is of a very basic level, the general English proficiency is low and unsatisfactory [36,37], and by itself is not sufficient for medical professional courses. English proficiency is considered as a part of the entry criteria to the medical schools. Despite English proficiency being a part of the weighted formula for entering medical schools, at present, there is no specific cutoff score or a minimal standardized English language proficiency requirement for entering a medical school in Thailand [38]. English as a foreign language, therefore, is a requirement for students at our medical school [33]. Once students are admitted, they begin enrolling in English courses starting in their first year of study. During medical school, students are required to complete 6 English courses. Four general English courses are taken during the first 3 years of medical school and are taught by nonmedical staff from the university’s language institute. They focus on foundational grammar, vocabulary, and communication skills, providing a general linguistic foundation for academic and social contexts. Two EMP courses are introduced in the second and third years of medical school. The content focuses on medical terminology and language skills necessary for academic and clinical tasks, such as reading medical literature, writing reports, and engaging in clinical communication. The curriculum shifts from general language acquisition in the initial years to specialized language skills tailored to medical contexts as students advance in their studies.

Annual feedback from our students consistently indicates that they perceive limited relevance of general English to their medical studies. This perception may stem from several factors. Despite years of English education, many students struggle with basic speaking skills due to insufficient vocabulary and grammar knowledge [39]. Additionally, a lack of intrinsic motivation often leads students to view general English as a mandatory requirement rather than a valuable skill for personal or professional development [40]. The culture within medical programs often emphasizes the need for English proficiency tailored to medical contexts, which shapes students’ perceptions and priorities [41]. Therefore, they could not match learning general English in the medical curriculum.

The findings from our students’ needs assessments revealed that our medical students needed to improve their English proficiency and wanted the school to organize a test for their English proficiency [42]. They preferred a self-directed web-based learning method and teachers who were both English language experts and medical professionals [42]. Based on the needs assessments, students value medical English because it directly aligns with their academic and professional goals. Unlike general English, medical English equips them with the specific skills needed to read medical literature, participate in clinical discussions, and engage effectively within global medical communities [41,43]. Needs assessments and feedback consistently emphasize students’ preference for EMP courses over general English, highlighting the practical benefits they associate with EMP in their medical studies and future careers. Based on learning needs assessments, 2 additional EMP courses were developed while 2 general English courses were removed from the medical curriculum. The newly developed EMP courses were English for Medical Purposes I for the second-year medical students and English for Medical Purposes II for the third-year medical students, which were launched at the same time in the academic year 2021.

Proficiency in medical English is considered to enhance students’ academic performance, evidenced by a positive correlation between English proficiency and academic success among medical students [44,45], and to support their development as independent learners [15]. However, evaluating the extent to which students achieve independent learning is beyond the scope of this study.

## Methods

### Study Objectives

This study aims to evaluate the relevance, acceptance, and satisfaction of structured web-based EMP courses among preclinical medical students.

### Study Design

A cross-sectional study was conducted to explore medical students’ opinions on learning EMP in a structured web-based course, and qualitative insights from instructors’ feedback were obtained.

### Participants

The participants were second-year medical students enrolled in the English for Medical Purposes I course and third-year medical students enrolled in the English for Medical Purposes II course at the time of the academic year 2021.

The WinPepi, a statistical software package, was used to calculate sample size based on the proportion of medical students who acknowledged web-based learning [46]. Assuming a proportion of 0.73, a population size of 535, a design effect of 1, and a significance level of 0.05, a total number of 121 was sufficient. However, all 535 students were included in this study to avoid a potential source of selection bias. It is important to clarify that this power analysis was performed for the broader research project, which consists of 2 parts. The first part, detailed in this manuscript, explores medical students’ perceptions and experiences with web-based EMP learning. The second part examines the effects of the structured web-based EMP courses on changes in students’ English language proficiency after course completion, which is reported in a separate article.

### Development of Structured Web-Based EMP Courses

Based on our medical students’ needs, EMP course objectives were set accordingly. The main objective of the EMP course was to enhance students’ medical English proficiency in all 4 core English language skills (reading, writing, listening, and speaking) through structured, targeted, and interactive web-based practice.

An overview of the four main English language skills is as follows. (1) Reading: Defined as the comprehension of medical literature and academic texts relevant to the students’ year of medical study. Instructional strategies include guided analysis of medical articles, scenario-based reading exercises, and formative assessments such as quizzes. (2) Writing: Focuses on effective medical note-taking and medical essays, emphasizing medical content, structure, vocabulary, readability, precision, and clarity. Instructional strategies involve structured writing guides, assignments, peer reviews, and scenario-based tasks such as summarizing patient cases. (3) Listening: Aimed at developing comprehension of medical conversations from case studies and lectures. Instructional methods include exposure to scenario-based audio materials, repeated listening tasks, and interactive multimedia resources. (4) Speaking: Enhances verbal communication in medical contexts, including explaining medical terms, conducting history taking, discussing treatment plans, and educating patients. Instructional strategies feature medical speaking guides, public speaking guides, role-play, video-recorded practice sessions, and feedback loops. Performance improvement for each skill is measured through targeted assessments conducted before and after the course. Standardized testing for reading and listening comprehension, comparison of baseline and final writing samples to assess coherence and technical accuracy, and evaluations of verbal communication in simulated scenarios are used to document and demonstrate progress effectively. These details ensure that the EMP course objectives are transparent and tied directly to improving students’ proficiency in medical English through targeted and practical methods.

To develop an effective structured web-based course, we followed the steps of Hays and Veitch’s recommendations [47] and applied Cook and Dupras’s guide [48]. Key principles of Hays and Veitch emphasize the importance of conducting a thorough needs assessment to identify gaps in knowledge, skills, and practices while ensuring relevance to participants’ professional roles and daily practices. They recommend interactive methods such as case studies, group discussions, and workshops to foster engagement and promote practical learning. Flexible delivery formats accommodate diverse learning preferences and schedules, while regular program evaluations ensure continuous improvement, sustainability, and adaptability to emerging needs and advancements in medical education. Cook and Dupras provide a guide specifically for developing web-based learning programs. Their framework focuses on conducting needs assessments, defining clear and measurable learning objectives, and designing content tailored to learners’ needs. They highlight the importance of interactive and multimedia-rich content to enhance engagement, as well as ensuring usability and accessibility for diverse learners. Evaluation methods, including pre- and postassessments, combined with continuous feedback and iterative improvements, are essential to measure learning outcomes and maintain program quality. Together, these frameworks provide a robust foundation for creating effective, learner-centered programs that meet educational goals and align with the needs of preclinical medical students. Barriers to web-based learning were considered [49]. More details regarding the development of EMP courses are provided in Multimedia Appendix 1.

The EMP courses were uploaded to a customized web-based learning management system (LMS) developed by our medical school [29]. Each EMP course ran throughout the semester for approximately 48 weeks, and the students’ learning schedule was approximately 3 hours per week. However, students can manage their time to study as scheduled or at any time that suits them (see more details on developing the web-based EMP course in Multimedia Appendix 1). Data from the LMS monitoring system were used to report student enrollment and completion of the EMP courses.

### Data Sources, Questionnaire, and Assessment

To evaluate the course, questionnaires were used as tools to gather student perspectives on various aspects of the courses, including their confidence in medical English skills, satisfaction with the content, and perceived benefits of web-based learning. These tools provided quantitative data on student experiences and attitudes. Additionally, feedback from instructors on course design and delivery was integrated into the evaluation process to ensure alignment with educational objectives and identify areas for improvement. Together, these methods offered both quantitative and qualitative insights into the evaluation of the web-based EMP courses.

An online administered questionnaire was developed based on the literature on the use and the evaluation of web-based learning [46,50,51]. The following constructs of web-based learning effectiveness and attitudes toward the structured web-based EMP course were included in the questionnaire: individual learners, confidence in medical English skills, perceived factors influencing web-based participation, perceived satisfaction with content and instructional designs, perceived ease of use, perceived advantages and barriers to web-based learning, and perceived outcomes and benefits of web-based EMP courses. The questionnaire included a 5-point Likert scale ranging from disagree to agree, which was used to assess learners’ agreement with each item. For learners’ confidence in their medical English skills, the constructs included a 5-point Likert scale ranging from not confident to strongly confident. For the factors influencing web-based learning participation, the constructs included a 5-point Likert scale, ranging from no influence to influence. For learners’ perceived benefits of web-based EMP courses, the constructs included a 5-point Likert scale, ranging from not beneficial to strongly beneficial.

To ensure the face and content validity of the questionnaire, each item was evaluated thoroughly by 3 separate experts in the field of medical education at our school. Thirty students participated in a pilot test of the questionnaire, which led to revisions. The Cronbach α coefficient of the attitude and experience part of the questionnaire was 0.93.

The details of the course instructions, content, and materials are provided in Multimedia Appendix 1. The course media included PDF files, audio, videos, and external links for the downloads. The second-year medical students completed a 45-hour web-based asynchronous English for Medical Purposes I course. The third-year medical students completed a 45-hour web-based asynchronous English for Medical Purposes II course. The students would be considered to have completed the module if that module was accessed at least 50% of the total learning time for that module because the LMS has a double speed-up function for playing video or audio clips.

After participants completed the web-based EMP courses, they were invited to complete the questionnaire. A link to the online questionnaire on Google Forms was added at the end of each course. The students completed the questionnaire between December 2021 and April 2022. The questionnaire data were transferred from Google Sheets, and data from the LMS monitoring system were exported to Microsoft Excel. The data were then gathered and checked for completion before being transferred to IBM SPSS for Windows.

Of note, the summative assessment for medical English proficiency was conducted using the Khon Kaen University Medical English Test (KKUMET), which examined proficiency in listening, reading, writing, and speaking. Each component was assessed before and after completing the EMP courses. The baseline EMP proficiency of the participating students, as assessed before enrollment in the 2 EMP courses, revealed that more than two-thirds of the students were at beginner or intermediate levels. The instructional goal of these EMP courses was to enhance their proficiency to intermediate and advanced levels. This study focuses on highlighting final outcomes; detailed results of the EMP proficiency tests conducted before and after these web-based EMP courses are reported in a separate article.

### Statistical Analysis

Data from questionnaires were analyzed using IBM SPSS for Windows version 26.0. A pairwise deletion strategy was applied to handle the missing data. The demographic data were described using descriptive statistics. The participants’ responses on a 5-point Likert scale to a 42-item questionnaire on their web-based learning experiences were dichotomized by calculating the mean scores and SD. Mean scores of 3.5 and above were considered agreed upon.

### Ethical Considerations

This study was approved by the Human Research Ethics Committee of Khon Kaen University (project number: HE631465). Students were recruited through the researcher’s assistant, who invited volunteers to participate. Before completing the questionnaires, participants were informed that participation was voluntary and that they could drop out of the study at any time. They were informed that their opinions were important for enhancing the medical English courses and were therefore encouraged to express them. All students voluntarily agreed to participate without receiving compensation. The participants’ privacy and identity were protected, and confidentiality was assured in that no identifying information was asked. The study objectives were explained to the participants, and the study was conducted according to the academic ethical code.

## Results

### Participating Medical Student Demographics

A total of 535 medical students completed the web-based EMP courses, 452 of whom started and returned the completed questionnaires (response rate: 84.5%). Table 1 presents the participants’ demographic data. The numbers of male and female participants were relatively similar, as were the numbers of medical students each year.

**Table 1.** Demographic data of the participants.

Demographics	Values
Age (n=451), years	
Range	18‐24
Mean (SD)	20.37 (0.74)
Sex (n=452), n (%)	
Male	219 (48.5)
Female	222 (49.1)
Prefer not to say	11 (2.4)
Year of study (n=450)	
2	231 (51.3)
3	219 (48.7)

### Influence of Web-Based Learning Characteristics

Respondents rated the degree of influence of the characteristics of web-based learning on their participation in web-based EMP courses (Table 2). Convenience, flexibility, and accessibility were rated the highest, while facilitator interactions received lower ratings.

**Table 2.** Rating of the influence of web-based learning characteristics on participation in web-based EMP^a^ courses (n=452).

Characteristics of web-based learning	The degree of influence on adoption of web-based EMP courses, mean^b^ (SD)
The convenience of completion the courses at any time or place	4.82 (0.49)
Flexibility to complete and save small sections at a time	4.80 (0.56)
Easy to access the course content	4.60 (0.75)
Easy to use/complete the course	4.58 (0.71)
Access to other useful links and resources	4.53 (0.84)
Instant access to feedback and the right answers when completing quizzes	4.50 (0.85)
The quality of content	4.46 (0.75)
Accessibility to technical support if difficulties are encountered	4.35 (0.94)
The use of case-based information and discussion	3.99 (1.08)
Facilitator’s regular input/participation	3.77 (1.17)
The opportunity to communicate/interact with the facilitator	3.75 (1.14)

a EMP: English for medical purposes.

b Mean was calculated using a 5-point Likert scale ranging from 1 (strongly no influence), 2 (no influence), 3 (neutral), 4 (influence), and 5 (strongly influence).

### Confidence in Medical English Skills

Respondents’ confidence in their medical English skills is shown in Table 3. The participating medical students reported feeling confident about medical English reading, vocabulary, and listening skills but were not sure about their writing skills.

**Table 3.** The participant’s confidence in their medical English skills after completing the medical English courses (n=452).

Confidence in medical English skills after completing the modules	Values, mean^a^ (SD)
Medical English reading skill	4.11 (0.87)
The use of medical English vocabulary	4.04 (0.84)
Medical English listening skill	4.00 (0.89)
Applying professional-specific knowledge and skills in English	3.92 (0.91)
Medical English speaking skill	3.50 (1.05)
Medical English writing skill	3.46 (1.07)

a Mean was calculated using a 5-point Likert scale ranging from 1 (strongly not confident), 2 (not confident), 3 (not sure), 4 (confident), and 5 (strongly confident).

### Perceived Advantages, Barriers To, and Attitudes Toward Instructional Designs of Web-Based EMP Courses

The participants identified useful and beneficial aspects of web-based EMP courses. The top 3 highly rated participants agreed on the advantages of convenience, sufficient instructions, and clear and easy-to-understand content (Table 4). For the instructional designs of the web-based EMP courses, the participants agreed on the clarity and appropriateness of the overall design of the courses, including clear objectives and content, appropriate content, arrangement, instruction, media, delivery method, course assessment, and grading (Table 5).

**Table 4.** The participants’ web-based learning experience of the medical English courses (n=452).

	Values, mean^a^ (SD)
Advantages of the web-based medical English modules	
I was able to learn at any place	4.77 (0.59)
Overall, this web-based course provided me with adequate instruction	4.50 (0.85)
The content was easy to understand and clear	4.41 (0.80)
Overall, the course contents covered its objectives	4.30 (0.87)
I felt more comfortable learning in this web-based course than in the face-to-face session	4.19 (1.13)
Overall, the instruction I obtained from this web-based program was motivating	4.16 (0.99)
I knew how to contact the facilitator and the facilitator responses promptly to my questions	4.04 (1.02)
The web-based program fulfilled my learning needs to improve my medical English skills	3.93 (1.04)
If I had technical problems during participating in this program, I received adequate help with technical problems (n=66)^b^	3.30 (1.05)
Difficulties in accessing and completing the course	
There was too much basic, well-known information in the course	3.06 (1.14)
The module took too long to complete	3.03 (1.28)
I spent more time in access to a computer to access this web-based program	2.31 (1.46)
The internet connection was very slow	2.25 (1.42)
I spent more time in downloading external links	2.08 (1.35)
The course was not useful for me because I do not have adequate computer skills to complete the course	1.95 (1.41)

a Mean was calculated using a 5-point Likert scale ranging from 1 (Strongly disagree), 2 (disagree), 3 (neutral), 4 (agree), and 5 (strongly agree).

b When the participants were asked if they had technical problems during participating in this program, of the 452 participants, 66 (14.6%) had, while 386 (85.4%) had not.

**Table 5.** The participants’ rating on the web-based medical English course contents and instructional designs (n=452).

The web-based course contents and instructional designs	Values, mean^a^ (SD)
The course had clear learning objectives	4.48 (0.79)
The contents were complete, appropriate, and relevant to the objectives	4.41 (0.82)
The order of contents was arranged properly	4.41 (0.82)
The learning media (eg, audios, videos, and PDF files) were appropriate	4.39 (0.90)
Overall, the instructional design of the program was appropriate	4.38 (0.85)
The web-based teaching was appropriate	4.37 (0.86)
The course’s assessments (formative assessments on listening, reading, writing, and speaking) were appropriate	3.97 (1.05)
Grading criteria were appropriate	3.74 (1.07)

a Mean was calculated using a 5-point Likert scale ranging from 1 (Strongly disagree), 2 (disagree), 3 (neutral), 4 (agree), and 5 (strongly agree).

### Perceived Learning Outcomes

The participants agreed that their 4 main skills improved. They also believed that all types of teaching media and lectures benefited them (Table 6).

**Table 6.** The participants’ perceived outcomes to and benefits of the web-based medical English courses after the course completion (n=452).

	Values, mean (SD)
The participants’ perceived outcomes after the course completion^a^	
I improved my listening skills	4.37 (0.85)
I improved my reading skills	4.23 (0.90)
I gained knowledge on how to practice my medical English skills	4.21 (0.89)
I improved my writing skills	3.74 (1.15)
I improved my speaking skills	3.68 (1.21)
Benefits of the web-based medical English courses after the course completion^b^	
The audios to practice listening skills	4.45 (0.83)
The media for medical terms to learn medical terms and practice reading, speaking, and writing skills	4.36 (0.83)
The videos to practice reading, listening, and speaking skills	4.35 (0.91)
The lectures on listening and reading	4.34 (0.84)
The course was organized in the modules	4.28 (0.92)
The recommended reading articles to practice reading and writing skills	4.07 (0.99)
The lectures on scientific writing	4.07 (1.00)
The lectures on speaking	4.02 (1.04)

a Mean was calculated using a 5-point Likert scale ranging from 1 (strongly disagree), 2 (disagree), 3 (neutral), 4 (agree), and 5 (strongly agree).

b Mean was calculated using a 5-point Likert scale ranging from 1 (strongly not beneficial), 2 (not beneficial), 3 (neutral), 4 (beneficial), and 5 (strongly beneficial).

### Learning Behaviors and Engagement Patterns (Data From the LMS Monitoring System)

The second-year students (n=263) completed the learning module ranging from 0 to 7 modules (mean 6.54, SD 1.41). The total learning time ranged from 172 to 24,439 minutes, with a mean of 925.02 (SD 1759.80) minutes. Regarding students’ learning behaviors, 153 (58.2%) out of 263 students spent less than 10 hours on the course and often logged in to the course during the afternoon (1069/2993 logins, 35.72%) and evening hours (1005/2993 logins, 33.58%).

The third-year students (n=272) completed the learning module ranging from 0 to 7 modules (mean 6.61, SD 1.41). The total learning time ranged from 0 to 14,100 minutes, with a mean of 768.59 (SD 930.56) minutes. Regarding students’ learning behaviors, 136 (50%) students spent less than 10 hours on the course and often logged in to the course during the evening (1000/2962 logins, 33.76%) and morning hours (655/2962 logins, 22.11%).

### Improvements in Medical English Proficiency

The summative assessment of medical English proficiency was conducted using KKUMET, which evaluates listening, reading, writing, and speaking skills. Each component was assessed before and after the EMP courses. The results showed statistically significant improvements in students’ scores across all 4 skills, as well as in the total test scores (*P*<.001), underscoring the effectiveness of the courses. Additional details about the summative testing results are presented in a separate article.

### Instructor Feedback on Course Design and Implementation

During the development and implementation of the structured web-based EMP courses, all course instructors actively participated in providing feedback. This feedback was gathered through regular meetings, written evaluations, and iterative reviews of course content and delivery methods. Instructors emphasized the importance of aligning the courses with medical students’ academic and professional needs. Their insights directly influenced several key aspects of the course design.

First, scenario-based learning was integrated to make the content more applicable to clinical practice. Case-based scenarios were incorporated into reading and listening exercises to enhance the relevance and practicality of the material. Second, interactive assessments were adapted based on instructor feedback to emphasize practical application. These included the use of peer reviews for writing assignments and role-play activities for speaking exercises, allowing students to practice and refine their skills in realistic scenarios. Third, instructors highlighted the need for multimedia resources to create a more engaging learning experience. This led to the inclusion of audio clips, videos, and interactive tasks designed to cater to various learning preferences. Finally, adaptive scheduling was implemented to accommodate the heavy workloads of medical students. The courses were designed to be asynchronous, enabling students to learn at their own pace and manage their schedules effectively.

Instructors reported that the structured design of the courses, combined with the iterative feedback process, resulted in a cohesive program that effectively addressed students’ learning needs. Their ongoing involvement ensured that the courses remained both relevant and practical for medical students.

## Discussion

### Principal Findings

This study explored the perspectives of preclinical medical students on structured web-based EMP courses and evaluated their proficiency improvements. A total of 535 students enrolled in and completed the courses by the published due dates, of which 452 (84.5%) students completed the questionnaire. The high response rate can be attributed to several factors. The survey’s integration at the end of the web-based EMP courses ensured participants encountered it immediately after course completion, when their experiences were fresh and engagement levels were high [52]. The web-based format allowed flexibility, enabling participants to complete the survey at their convenience [53]. Clear instructions, the assurance of anonymity, and the perceived relevance of the survey further encouraged participation [54]. Additionally, students’ satisfaction with the course content and instructional design likely motivated them to provide feedback [52].

The summative assessment, conducted using KKUMET, revealed statistically significant improvements across all 4 English language skills (listening, reading, writing, and speaking). Participants reported high confidence in reading, vocabulary, and listening skills but expressed lower confidence in writing and speaking skills. Students rated convenience, clarity of content, and sufficient instruction as the top benefits of the courses. These results affirm the relevance, acceptance, and satisfaction of structured web-based EMP courses for medical students, aligning with the primary objectives of improving proficiency and fostering recognition of EMP’s importance in supporting their academic learning.

### Evaluation of Course Effectiveness

The primary goal of the EMP courses was to improve students’ proficiency in all 4 English language skills and introduce relevant medical terminology tailored to their academic content. This goal was achieved, as evidenced by measurable improvements in KKUMET scores and student-reported confidence levels.

We evaluated the effectiveness of web-based EMP courses, finding significant improvements in students’ medical English proficiency across all skills, as measured by KKUMET scores, and increased confidence in reading, vocabulary, and listening. By comparing subjective learner feedback with objective results, we confirmed that reported confidence aligned with measurable gains, minimizing potential overestimation from cognitive biases like the Dunning-Kruger effect [55,56]. These findings underscore the importance of validating self-reported outcomes with objective data to ensure reliable assessments of learning impact [48,57], with future research recommended to explore the influence of cognitive biases and interventions to enhance learning outcomes [55,58].

Furthermore, the courses aimed to highlight the importance of EMP in supporting learning across other subjects, particularly through contextual and content-based design [7,15]. Students expressed initial difficulty in understanding the importance of general English and its relevance within the medical curriculum. However, they highly valued the structured web-based EMP courses and expressed satisfaction with the content, particularly due to its alignment with their academic and professional needs. This alignment was achieved by designing and developing courses that were self-directed and created by medical professionals, which students recognized as enhancing their engagement and acceptance of the educational approach. While students did not explicitly indicate a need for increased interaction with faculty facilitators, they expressed a preference for instructors who were both English language experts and knowledgeable about medical content. This preference suggests an implicit desire for contextualized guidance, highlighting the value students place on instructors with dual expertise. The structured web-based EMP courses effectively met these needs, motivating them to integrate EMP learning into their study schedules, underscoring the importance of tailoring course design to align with student preferences.

### Comparison With Previous Studies

The findings of this study align with existing research on the effectiveness of web-based learning in medical education while addressing gaps specific to EMP. Prior studies have emphasized the effectiveness of web-based learning in enhancing knowledge and skills in medical education [23,24]. Similarly, this study demonstrated statistically significant improvements in all 4 core English language skills (listening, reading, writing, and speaking) among medical students enrolled in structured web-based EMP courses. These findings confirm that well-designed web-based educational modalities can be as effective as traditional methods.

Students greatly appreciated the flexibility and accessibility offered by web-based courses, a perspective consistently supported in previous research [25,26,29,30]. Students appreciated the ability to learn at their own pace, access course materials conveniently, and engage with multimedia content tailored to their needs. The use of diverse teaching media, such as audio, video, and interactive exercises, aligns with widely recognized recommendations for enhancing learning engagement and retention [59,60]. This highlights the role of self-directed learning in increasing engagement and satisfaction.

Unlike previous studies focusing on blended learning or face-to-face instruction [25-27], this study uniquely explored the exclusive use of structured web-based courses for EMP. The findings underscore the potential of this approach to address the challenges faced by medical students in non–English-speaking regions, thereby filling a critical gap in the literature.

This study also found that students reported greater confidence in receptive skills (reading and listening) than in productive skills (writing and speaking), a pattern consistent with findings from previous research [7,15,17]. This underscores the need for targeted instructional strategies to support the development of productive language skills in EMP courses [2,17].

By situating the findings within the broader body of research, this study contributes to the evolving understanding of effective teaching strategies for EMP and web-based learning in medical education.

### Implications of Findings for Practice

The findings of this study have important implications for curriculum development and teaching strategies in medical schools, particularly in non–English-speaking contexts. Structured web-based EMP courses significantly improved students’ medical English proficiency, demonstrating their potential to meet academic and professional needs effectively. These results suggest that similar approaches could be adopted by other medical schools to enhance student engagement and learning outcomes.

Medical schools can leverage the flexibility and accessibility of web-based learning to design EMP courses that accommodate students’ demanding academic schedules. By focusing on targeted skill development and incorporating medical terminology into course content, institutions can create tailored programs that address specific language needs [14,19]. The self-paced nature of web-based learning further enables students to manage their time effectively, aligning with their individual schedules and learning preferences. This adaptability can increase motivation and reduce barriers to learning, particularly in resource-constrained settings.

Integrating EMP with other areas of medical education, such as reading medical literature and writing clinical reports, can help students perceive medical English as an essential part of their academic and professional journey rather than as a standalone requirement. Additionally, structured web-based courses offer scalable and accessible solutions for institutions with large student cohorts, ensuring consistent, high-quality content delivery while reducing the resource burden on faculty and support staff.

Despite the benefits of self-directed learning, low engagement with “consultant hours” highlights the need for integrating opportunities for active faculty-student interaction within course designs. Addressing this issue could involve developing mechanisms that encourage and normalize faculty interaction, which may be especially beneficial in contexts where cultural preferences for independence or heavy academic workloads limit voluntary engagement with support services [23,24,26]. Such measures could enhance overall student support and satisfaction.

These insights underscore the value of structured web-based EMP courses as a model for improving language proficiency and supporting broader academic goals in medical education.

### Strengths and Limitations

This study offers a comprehensive evaluation of structured web-based EMP courses, combining subjective learner feedback with objective proficiency measures. A key strength lies in the design of the courses, informed by needs assessments and evidence-based guidelines [47,48]. Moreover, the inclusion of summative assessments provides robust evidence of the courses’ effectiveness. This study had a large sample size and response rate, which ensures robust findings. However, the study’s scope was limited to a single medical school, potentially affecting generalizability [11,57].

Future research should focus on strategies to enhance productive skills (writing and speaking) while continuing to explore the impact of cognitive biases, such as the Dunning-Kruger effect, on the gap between perceived and actual proficiency [56]. Large-scale, multi-institutional studies are warranted to validate these findings and provide broader recommendations for integrating EMP into medical curricula. Additionally, research should investigate the long-term impact and scalability of such courses across diverse educational settings, as well as the development of adaptive learning technologies to customize course content based on students’ baseline proficiency levels, effectively addressing specific skill gaps.

### Conclusions

Structured web-based EMP courses are highly relevant, widely accepted, and well-received by medical students, demonstrating significant improvements in their medical English proficiency, particularly in reading, vocabulary, and listening skills, as evidenced by both subjective feedback and objective measures. The flexibility, accessibility, and practicality of structured web-based learning make it an effective approach to address the unique challenges faced by medical students with demanding schedules. By tailoring course content to meet students’ academic and professional needs and incorporating engaging instructional designs, these courses provide a scalable and sustainable solution for medical education in non–English-speaking regions. Future developments in EMP course design should focus on enhancing productive language skills, such as writing and speaking, while maintaining the balance between self-directed learning and faculty support, integrating these courses into medical curricula as an essential component to equip students with the language skills necessary for academic success and global medical practice.

## Supplementary material

10.2196/65779 Multimedia Appendix 1 Development of structured web-based English for medical purpose courses.

## Abbreviations

EMP: English for medical purposes
KKUMET: Khon Kaen University Medical English Test
LMS: learning management system
TCAS: Thailand University Central Admission System
