# Supplementary material for: Preclinical Medical Students' Perspectives and Experiences With Structured Web-Based English for Medical Purposes Courses: Cross-Sectional Study
Source: JMIR Med Educ. 2025 Mar 27;11:e65779. doi: 10.2196/65779 (PMC11967694; doi:10.2196/65779)
Supplement: Multimedia Appendix 1 [file mededu-v11-e65779-s001.docx]

**Multimedia Appendix**

This Multimedia Appendix is provided for additional information regarding the development of structured online English for Medical Purpose courses.

**Development of Structured Online English for Medical Purpose (EMP) Courses**

According to the students’ annual feedback on (general) English learning that they could not see its importance and usefulness in their medical studies, or its place in the medical curriculum. We have added these comments on the development of these two new EMP courses.

Based on the students’ feedback, the concepts of developing EMP courses included the importance, relevance, and advantages of the EMP to the students’ current medical studies. These were integrated into the course introduction as well as the course content. In addition, EMP courses should not add an additional burden to students learning other subjects in the medical curriculum. By contrast, EMP courses should support medical students in learning other subjects in the curriculum. Therefore, the key point is that learning EMP courses should be flexible and convenient. Flexibility means completing and saving a small section at a time at one individual’s pace, and convenience means completing or learning the course at any time or location. This concept falls into the principle of structured online learning, in which this educational method was also the preferred method for our students according to the results of the students’ needs assessment [1]. The EMP courses were designed based on this concept. The method of delivery used to serve students’ needs is a structured online course.

Barriers to online learning were also considered prior to the development of these online EMP courses. Evidence suggests that barriers to online learning can be classified into four main groups [2]: structure-related difficulties (internet connections and technical difficulties), learner- or participant-related factors (lack of computer knowledge and skills, lack of time and peer interaction, attitudes and preferences toward online learning), facilitator-related difficulties (insufficient support in developing online courses, lack of facilitating skills, attitudes toward online teaching methods), and factors related to the online program (ease of use and completion, design and quality of the content).

To develop an effective structured online course, we followed the steps of Hays and Veitch [3] and applied Cook and Dupras’s guidelines [4]. Based on these guidelines, we obtained students’ needs through annual feedback and evaluation of the course, and from students’ needs assessment [1]. We then set objectives for the courses according to the learners’ needs to improve their proficiency in all four main skills: reading, listening, writing, and speaking. Thus, each of the four skills was tested before and after the completion of the EMP course. Notably, vocabulary learning was included in each of the four main skills. In addition, the online EMP courses were tested for their effectiveness on preclinical medical students’ medical English proficiency and their relationships with the students’ academic performance and success, which will be published elsewhere.

The main objective of the EMP course was to enhance students' medical English proficiency in all four core English language skills (reading, writing, listening, and speaking) through structured, targeted, and interactive online practice. A detailed overview of the four main English language skills as follows:

Reading: Defined as the comprehension of medical literature and academic texts relevant to the students' year of medical study. Instructional strategies include guided analysis of medical articles, scenario-based reading exercises, and formative assessments such as quizzes. Performance improvement measures incorporate pre- and post-course standardized reading assessments to evaluate students' progress.

Writing: Focuses on effective medical note-taking and medical essay, emphasizing medical content, structure, medical vocabulary, readability, precision and clarity. Instructional strategies involve structured writing guides, assignments, peer reviews, and scenario-based tasks like summarizing patient cases. Performance improvement measures include comparison of baseline and final writing samples to assess gains in coherence and technical accuracy.

Listening: Aimed at developing comprehension of medical conversations from case studies and lectures. Instructional methods include exposure to scenario-based audio materials, repeated listening tasks, and interactive multimedia resources. Improvement is measured through listening comprehension tests conducted at the beginning and end of the course.

Speaking: Enhances verbal communication in medical contexts, including explaining medical terms, conducting history taking, discussing treatment plans, and educating patients. Instructional strategies feature medical speaking guides, public speaking guides, role-play, video-recorded practice sessions, and feedback loops. Speaking skill improvement is measured through performance evaluations in simulated scenarios.

In alignment with these definitions, instructional strategies such as scenario-based exercises, multimedia resources, and formative assessments have been elaborated. Furthermore, performance improvement measures, including pre- and post-course standardized testing for each skill, have been outlined to document and demonstrate progress effectively. These details ensure that the EMP course objectives are transparent and tied directly to improving students' proficiency in medical English through targeted and practical methods.

Therefore, we designed the course so that learners could practice all skills. The course content was based on the content of the medical curriculum for that particular year of medical study. As a result, these EMP contents were related to the curriculum and context of preclinical studies and the Thai healthcare system, and we designed instructions for students to practice English skills based on these contents and contexts. To do so, it not only supports students in learning other compulsory subjects by becoming familiar with the medical terms of those subjects through learning in this EMP course, learning how to effectively read and note taking in those areas, but also learning through clinical scenarios how these basic medical sciences could apply to clinical situations.

The contents of the English for Medical Purposes I course included anemia, gastritis, diabetes, and type 2 diabetes, which were selected based on the topics the medical students learned in the second year. Each topic was added to the lessons, which were organized into modules. The modules include history taking, physical examination, diagnosis, treatment, patient education, and discussion of health-related topics. Before starting the module, guides to practice medical English skills were provided, including what medical English is, listening and reading skills, scientific writing, and speaking. Each module was arranged in the following order: 1) module’s guide (what and how to learn in the module), 2) audio of the topic (listening to the conversations in the scenario), 3) medical terms of the topic (learning new vocabulary and medical terms from the scenario), 4) animation of the topic (listening again through animated video with subtitles in the same scenario as in the audio), 5) reading resources of the topic (reading research articles related to the topics), 6) writing exercises on the topic (self-practicing based on the reading articles or writing an explanation of the medical terms), and 7) speaking exercises on the topic (self-practicing based on the conversations of the scenario). This order was arranged and repeated for each topic to form lessons for each module. For the English for Medical Purposes I course, four selected topics were generated in 21 scenarios (animated videos) (see Table S1).

**Table S1.** List of courses, clips, and duration of English for Medical Purposes I.

| **Module** | **Topics** | **Instruction** | **Media** | **Duration^a^ (minutes)** |
| --- | --- | --- | --- | --- |
| Module 1: Guides to practice medical English skills | 1. What is “Medical English?”  2. Listening and reading skills  3. Scientific writing  4. Speaking | Listing to 4 lectures as mentioned topics | 4 lecture videos | - |
|  | Total: 4 lessons and 4 clips for Module 1 | | | 42.8 |
| Module 2: History taking | 4 medical topics | Lesson 1: Introduction of what will learn in this module. | pdf | - |
|  | 1) Anemia | Lesson 2: Listening audio: anemia | audio | 4.20 |
|  |  | Lesson 3: Medical terms | Script (pdf) | - |
|  |  | Lesson 4: Listening animation: anemia | Animated video | 4.20 |
|  |  | Lesson 5: Reading resources: anemia | List of medical article relevant to anemia | - |
|  |  | Lesson 6: Writing exercise: anemia | Taking notes of new medical terms of the topic based on script or summrizing the articles and medical essay | - |
|  |  | Lesson 7: Speaking exercise: anemia | Practicing based on medical speaking guides by imitating the conversations from the animated video; and by explanations of medical terms and small talk of illnesses relevant to anemia | - |
|  | 2) Gastritis | Repeating sequence of instructions and medias like anemia from Lesson 2 to 7 | | 14.26 |
|  | 3) Diabetes | Repeating sequence of instructions and medias like anemia from Lesson 2 to 7 | | 7.55 |
|  | 4) Type 2 diabetes | Repeating sequence of instructions and medias like anemia from Lesson 2 to 7 | | 5.06 |
|  | Total: 25 lessons and 8 clips for Module 2 | | | 35.27 |
| Module 3: Physical examination | 3 medical topics (Thalassemia, gastritis and diabetes) and having sequences of 7 lessons like Module 1. Each medical topic would repeat instructions and medias from Lesson 2 to 7 like in Module 2 | | |  |
|  | Total: 19 lessons and 6 clips for Module 3 | | | 23.47 |
| Module 4: Diagnosis | 3 medical topics (Thalassemia, gastritis and diabetes) and sequences of 7 lessons like Module 1. Each medical topic would repeat instructions and medias from Lesson 2 to 7 like in Module 2 | | |  |
|  | Total: 19 lessons and 6 clips for Module 4 | | | 25.68 |
| Module 5: Treatment | 3 medical topics (Thalassemia, gastritis and Type 2 diabetes) and sequences of 7 lessons like Module 1. Each medical topic would repeat instructions and medias from Lesson 2 to 7 like in Module 2 | | |  |
|  | Total: 19 lessons and 6 clips for module 5 | | | 27.38 |
| Module 6: Patient education | 4 medical topics (Thalassemia, gastritis and diabetes Part I and Part II) and sequences of 7 lessons like Module 1. Each medical topic would repeat instructions and medias from Lesson 2 to 7 like in Module 2 | | |  |
|  | Total: 25 lessons and 8 clips for module 6 | | | 34.2 |
| Module 7: Discussion in health related topics | 4 medical topics (Medical appointment, repeat prescription, International Classification of Disease (ICD) and skin cancer) and sequences of 7 lessons like Module 1. Each medical topic would repeat instructions and medias from Lesson 2 to 7 like in Module 2 | | |  |
|  | Total: 25 lessons and 8 clips for module 7 | | | 28.7 |
| Total | The course has 7 modules, 136 lessons and 46 clips | | | 217.5 |

Note. ^a^Duration was calculate from duration time of only audios and animated videos.

The contents of the English for Medical Purposes II course included the cardiovascular system, chronic diseases, women’s health, men’s health, respiratory system, urinary system, and patient education. These systems were selected based on the ones that medical students learned in their third year. Under each system, we selected three medical conditions/diseases to create a scene. The course design was similar to that of the English for Medical Purposes I course, that is, for each topic, the students practiced all skills. For the English for Medical Purposes II course, seven selected systems were generated into 27 scenarios (animated videos) (see Table S2).

**Table S2.** List of courses, clips, and duration of English for Medical Purposes II.

| **Modules** | **Topics** | **Instructions** | **Medias** | **Duration^a^ (minutes)** |
| --- | --- | --- | --- | --- |
| Module 1: Guides to practice medical English skills | 1. What is “Medical English?”  2. Listening and reading skills  3. Scientific writing  4. Speaking | Listing to 4 lectures as mentioned topics | 4 lecture videos | - |
|  | Total: 4 lessons and 4 clips for Module 1 | | | 42.8 |
| Module 2: Cardiovascular system | 3 medical topics: | Lesson 1: Introduction of what will learn in this module. | pdf | - |
|  | 1) Heart failure | Lesson 2: Listening audio: case-based learning physiology-pathology of health failures | audio | 5.38 |
|  |  | Lesson 3: Medical terms: case-based learning physiology-pathology of health failures | Script (pdf) | - |
|  |  | Lesson 4: Listening animation: case-based learning physiology-pathology of health failures | Animated video | 5.37 |
|  |  | Lesson 5: Reading resources: heart failure | List of medical articles relevant to anemia | - |
|  |  | Lesson 6: Writing exercise: heart failure | Taking notes of new medical terms of the topic based on script or summarizing the articles and medical essay | - |
|  |  | Lesson 7: Speaking exercise: heart failure | Practicing based on medical speaking guides by imitating the conversations from the animated video; and by explanations of medical terms and small talk of illnesses relevant to heart failure | - |
|  | 2) Ischemic heart diseases | Repeating sequence of instructions and medias like heart failure from Lesson 2 to 7 | | 8.36 |
|  | 3) Dyslipidemia | Repeating sequence of instructions and medias like heart failure from Lesson 2 to 7 | | 10.30 |
|  | Total: 19 lessons and 6 clips for module 1 | | | 29.41 |
| Module 3: Chronic diseases | 3 medical topics (Diabetes, hypertension, osteoarthritis) and having sequences of 7 lessons like Module 1. Each medical topic would repeat instructions and medias from Lesson 2 to 7 like in Module 2 | | |  |
|  | Total: 19 lessons and 6 clips for Module 2 | | | 30.37 |
| Module 3: Women health | 3 medical topics (Contraception, pregnancy planning and menopause) and sequences of 7 lessons like Module 1. Each medical topic would repeat instructions and medias from Lesson 2 to 7 like in Module 2 | | |  |
|  | Total: 19 lessons and 6 clips for module 3 | | | 37.65 |
| Module 4: Men health | 3 medical topics (Sexual transmitted infection, erectile dysfunction and benign prostatic hyperplasia) and sequences of 7 lessons like Module 1. Each medical topic would repeat instructions and medias from Lesson 2 to 7 like in Module 2 | | |  |
|  | Total: 19 lessons and 6 clips for Module 4 | | | 36.94 |
| Module 5: Respiratory system | 3 medical topics (Upper respiratory tract infection, pulmonary tuberculosis and chronic obstructive pulmonary disease) and sequences of 7 lessons like Module 1. Each medical topic would repeat instructions and medias from Lesson 2 to 7 like in Module 2 | | |  |
|  | Total: 19 lessons and 6 clips for Module 5 | | | 45.18 |
| Module 6: Urinary system | 3 medical topics (Urinary tract infection, chronic kidney disease and nephrolithiasis—renal calculi) and sequences of 7 lessons like Module 1. Each medical topic would repeat instructions and medias from Lesson 2 to 7 like in Module 2 | | |  |
|  | Total: 19 lessons and 6 clips for Module 6 | | | 38.16 |
| Module 7: Patent education | 9 medical topics (Cardiovascular disease risk factors, how to lose weight, lowering cholesterol level, quitting smoking, arthritis, erectile dysfunction, a combined pill, pregnancy planning, asthma) and skin cancer) and sequences of 7 lessons like Module 1. Each medical topic would repeat instructions and medias from Lesson 2 to 7 like in Module 2 | | |  |
|  | Total: 55 lessons and 18 clips for Module 7 | | | 76.00 |
| Total | The course has 8 modules, 173 lessons and 58 clips | | | 336.51 |

Note. ^a^Duration was calculate from duration time of only audios and animated videos.

The course-development team included academic staff and production house teams. The academic staff were all from medical (MD) backgrounds and had successfully completed their master’s and doctoral degrees in international programs. In addition, one of the academic staff members completed and qualified the Teaching English to Speakers of Other Languages (TESOL) course from a country where English is an official language. The EMP course committees were academic staff with both MD and non-MD backgrounds, some of whom taught preclinical medical students, and the majority taught both preclinical and clinical medical students. The steps involved in developing each scenario for the animated videos are listed in Table S3.

**Table S3.** Steps in developing each teaching animated video.

| **Step** | **Details** |
| --- | --- |
| 1 | Writing script for the scenario by RM (the framework included two preclinical medical students discussing the topic they learned, doctor-patient communication, and the doctor talking with a colleague). The conversations would take less than 10 minutes (most videos took 5-6 minutes). |
| 2 | Checking the script for its accuracy, appropriateness in the context of Thai medical education and healthcare system, and relevance to Thai general practice using holistic approach/diagnosis/care by IT. |
| 3 | RM revised the scripts after step 2. |
| 4 | The script in step 3 would then send to the production house team to make the sound (sound was generated by the program). |
| 5 | RM and IT check the sound for pronunciation. If there was any error, it would send back to the production house to revise until the sound was accurate and appropriate. |
| 6 | The production house selected cartoon images, made background scenes, organizing the facial expressions and gestures of cartoon characters. |
| 7 | RM and IT checked the accuracy of the facial expressions and gestures of the characters to suit the dialogue and also related scene images and environmental elements. If there is any change, the production house team would revise it until approval. |
| 8 | The production house added subtitles into the video. RM and IT checked the accuracy of the subtitles. |
| 9 | The media was then uploaded to our online platform in two separate media, i.e audio (only sound) and animated video. |

For these two EMP courses, it took approximately one year for the team to complete course production. The courses were uploaded to a customized online learning management system (LMS) developed by our medical school. The courses were pilot tested prior to launch, and course committees were invited to enroll in the courses. Final checks and revisions were performed. Both courses were launched simultaneously in the second semester of the academic year 2021. Each EMP course ran throughout the semester for approximately 48 weeks, and the students’ learning schedule was approximately three hours per week. However, students can manage their time to study as scheduled, or at any time that suits them.

In summary, the specific technologies used in the online EMP courses included:

1. Interactive Elements:

- Scenario-based simulations were used to engage students and reinforce learning outcomes.
- Each module featured activities that allowed students to practice listening, reading, writing, and speaking skills in a structured sequence.

2. Video Production Techniques:

- Instructional videos provided integrated subtitles for accessibility.
- Animated scenarios were designed using a production house specializing in educational media. These scenarios included medical conversations (e.g., doctor-patient and peer discussions) and were developed following a 9-step production process (outlined in Table S1).

3. Steps in Animated Video Development:

- The scripts for animated videos were developed collaboratively by academic staff with medical and TESOL expertise.
- Quality assurance was ensured at multiple stages, including script accuracy, sound pronunciation, and visual appropriateness (e.g., facial expressions, gestures, and subtitles).

4. Learning Management System (LMS):

- The courses were hosted on a customized LMS developed by the medical school. Features included: A modular structure for self-paced learning; embedded multimedia content (audio files, videos, and PDFs); and progress tracking and self-assessment tools.
- Flexibility was emphasized, allowing students to complete modules at their convenience.

5. Content Relevance:

- EMP courses were aligned with the medical curriculum for the second and third years, featuring topics such as anemia, diabetes, cardiovascular health, and respiratory diseases.
- Modules were structured to introduce medical terminology, facilitate guided listening and reading, and culminate in writing and speaking exercises.

**References**

1. Suwanrot K, Sausukpaiboon K, Ketdao N, et al. Learning needs assessment for english language of medical students and residents in Srinagarind Hospital, Faculty of Medicine, Khon Kaen University. *Srinagarind Med J*. 2017;32(5):454-460.
2. Thepwongsa I. Education of rural and remote general practitioners (GPs) in Australian on type 2 diabetes : impact of online continuing medical education on GPs’ knowledge, attitudes and practices and barriers to online learning. Thesis. Monash University; 2017. <https://doi.org/10.4225/03/58ae4580ba641>
3. Hays R, Veitch C. Continuing medical education and divisions. In: Hays R (Richard B, Veitch C, editors. Contin Med Educ Gen Pract / Ed by Richard Hays, Craig Veitch Townsville, Australia: School of Medicine, James Cook University; 1999. p. 5–17.
4. Cook DA, Dupras DM. A practical guide to developing effective web-based learning. *J Gen Intern Med*. Jun 2004;19(6):698-707. PMID:15209610
